# The future of global health partnerships: Co-investment, reciprocal learning and mutual benefit

**DOI:** 10.1371/journal.pgph.0007017

**Published:** 2026-08-12

**Authors:** Ben Simms, Jim Campbell, Margaret Caffrey, Moses W. Mulimira, Titilola Banjoko Osiyemi, Navina Evans

**Affiliations:** 1 Global Health Partnerships, London, United Kingdom; 2 King’s Population Health Institute, King’s College London, London, United Kingdom; PLOS: Public Library of Science, UNITED STATES OF AMERICA

## Introduction

Global health is being reshaped by a workforce crisis that no single country can resolve on its own. High-income health systems recruit internationally at growing scale [1] even as the countries that trained those staff face their own shortages; official development assistance for health budgets that once drove investments to mitigate the impact of this mobility are projected to decline by 29–46% from 2024 to 2026 [2]; and the many debates on the future of global health and development assistance [3,4] recognise that international spending commitments on ‘aid’ and ‘assistance’ are of a bygone era.

## Honesty and humility

These themes took centre stage at the UK Global Health Summit, held in London from 16 to 18 March 2026, bringing together more than 600 participants from 28 countries, including serving Health Ministers, UK parliamentarians, and diaspora health professionals. The Summit’s opening address set out two organising principles for everything that followed: *honesty* about what the UK gains from a globally engaged health workforce, and *humility* about what the UK’s health system can learn from its partners in the global South [5].

The honesty half of that argument rested on a single, hard-to-dispute fact: more than one in five NHS staff report a non-British nationality [6], and a new cross-party UK parliamentary report launched at the Summit - entitled ‘An honest account of the benefits and costs of international health worker recruitment’ - put the UK’s saving in education costs averted at £14 billion [7]. Dr Iziaq Adekunle Salako, Nigeria’s Federal Minister of State for Health and Social Welfare, gave the same phenomenon a Nigerian dimension: over half of his country’s doctors have taken concrete steps toward emigration, and the education losses incurred from each departing physician exceed $200,000 [5]. Unlike the Robin Hood tax model, when it comes to health worker migration the global north is “the rich, who take from the poor” [5].

The second principle on humility was evident in the bi-directional benefits of UK-Ukraine and UK-Uganda health partnerships. Collaboration between The Christie NHS Foundation Trust in Manchester, Europe’s largest single-site cancer centre, and the National Cancer Institute of Ukraine in Kyiv, has brought Ukrainian surgeons and oncologists to Manchester to train in advanced surgical and radiotherapy techniques [8]. But the exchange does not run in one direction. Ukrainian clinicians, treating cancer patients under conditions of active war - disrupted radiotherapy infrastructure, patient pathways broken by occupation and missile strikes - have taught their UK counterparts about clinical adaptation and efficiency under extreme pressure [5]. As one Christie clinician put it, the project has been “about bilateral learning, as any partnership should be” [5]. In addition, Dr Diana Atwine, Uganda’s Permanent Secretary of Health, offered an insightful formulation of the same shift: partnership had moved from what she called *osmosis*, in which everything flows one way, to *equilibrium*, in which “everyone has something to bring on board” [5].

That humility is not a hypothetical standard for the UK Government to meet; it is one the Government has invested in through 18 years of continuous funding for health partnerships. The Summit showcased the UK Government-funded Global Health Workforce Programme, through which over 600 UK health workers volunteered, building the capacity of some 30,000 health workers through training, leadership development and system strengthening initiatives across six countries, aligned with national workforce priorities [5].

## Reflections

In a process of reflection on the UK Global Health Summit two points stand out.

Firstly, its convergence with the inaugural African-Nordic Health Summit, convened in Stockholm in January 2026, and reported by Jensen et al in this journal in May 2026 [9]. The two Summits exhibit different institutional histories, geographies and thematic entry points yet both concluded that the donor-recipient framing of development assistance had become descriptively false rather than merely outdated, and both replaced it with a reciprocity argument grounded in structural interdependence rather than the moral appeal of aid. Neither summit was responding to the other. That the two processes, run independently, arrived at a similar reframing is a stronger form of evidence than either could produce alone. It suggests that interdependence and bi-directional learning are now embedded within a new contemporary generation of global health partnerships.

Secondly, Dr Beccy Cooper MP, chair of the All-Party Parliamentary Group responsible for the ‘Honest Account’ publication, argued that the pandemic had already demonstrated a point governments had yet to fully grasp: “how interdependent we actually are” - not least in the health workforce - and what was needed now was “an era of responsibility” matched by meaningful co-investment and skills partnerships [10]as set out in the revision to WHO’s Global Code of Practice on the International Recruitment of Health Personnel [11]. The UK Government’s own actions in the following months bears that responsibility argument out: co-hosting the Global Partnerships Conference with South Africa in May 2026 [12], and launching a Global Partnerships Compact with an emphasis on strong, respectful partnerships that enable country and community leadership, and support skills development, learning and mutual benefit [13]. And in July 2026, Mr Andy Burnham MP, the UK’s incoming Prime Minister, presented a statement that: “In an increasingly interconnected world, the challenges we face are global and require global solutions. Britain should show leadership on the international stage, not retreat from it”.[14]

## Future of global health partnerships

We are therefore optimistic for the future of global health partnerships. A contemporary model for international cooperation is evolving. Three partnership events reaching similar conclusions without coordination is both a signal of a new momentum and fortuitous timing as the future of global health debate unfolds and the UK Government prepares to chair the G20 in 2027 [15]and the G7 in 2028.

Having helped shape the UK Global Health Summit, and organised the Global Partnerships Conference that followed, and having already funded the kind of reciprocal partnerships its own Compact describes, the UK Government now has the evidence, the credibility and, in its G20 and G7 presidencies, the platforms to coalesce international cooperation around a new generation of global health partnerships: an era premised on honesty and humility and with intentional priority on reciprocal learning and mutual benefit.
